# KCNF1 promotes lung cancer by modulating ITGB4 expression

**DOI:** 10.1038/s41417-022-00560-4

**Published:** 2022-11-16

**Authors:** Ching-Yi Chen, Pei-Ying Wu, Michelle Van Scoyk, Stephanie A. Simko, Chu-Fang Chou, Robert A. Winn

**Affiliations:** Massey Cancer Center, Virginia Commonwealth University, Richmond, VA USA

**Keywords:** Non-small-cell lung cancer, Molecular biology

## Abstract

Lung cancer continues to be the leading cause of cancer death in the United States. Despite recent advances, the five-year survival rate for lung cancer compared to other cancers still remains fairly low. The discovery of molecular targets for lung cancer is key to the development of new approaches and therapies. Electrically silent voltage-gated potassium channel (KvS) subfamilies, which are unable to form functional homotetramers, are implicated in cell-cycle progression, cell proliferation and tumorigenesis. Here, we analyzed the expression of KvS subfamilies in human lung tumors and identified that potassium voltage-gated channel subfamily F member 1 (KCNF1) was up-regulated in non-small cell lung cancer (NSCLC). Silencing of KCNF1 in NSCLC cell lines reduced cell proliferation and tumor progression in mouse xenografts, re-established the integrity of the basement membrane, and enhanced cisplatin sensitivity. KCNF1 was predominately localized in the nucleoplasm and likely mediated its functions in an ion-independent manner. We identified integrin β4 subunit (ITGB4) as a downstream target for KCNF1. Our findings suggest that KCNF1 promotes lung cancer by enhancing ITGB4 signaling and implicate KCNF1 as a novel therapeutic target for lung cancer.

## Introduction

Lung cancer commonly diagnosed in both men and women continues to be the leading cause of cancer death in the United States [1]. More than half of all patients with lung cancer will present at an advanced stage at the time of their diagnosis. Moreover, despite recent advances, the five-year survival rate for lung cancer compared to other cancers still remains fairly low [2]. The discovery of both molecular targets and immunotherapy treatments for lung cancer represents a significant advancement in the progress of “precision” oncology [2]. However, many of these current therapies may only be effective in some, but not all patients; thus, demanding the development of new approaches and therapies.

Potassium (K^+^) channels are diverse transmembrane proteins that selectively facilitate the permeation of K^+^ between intracellular and extracellular environments. There are four classes of K^+^ channels: voltage-gated, calcium-activated, inward-rectifier, and two-pore-domain K^+^ channels [3]. Voltage-gated K^+^ (Kv) channels are a diverse family of channels [4] that play crucial roles in both excitable and non-excitable cells. In excitable cells, they regulate resting membrane potential, and the shape and frequency of action potentials [5–7]. These functions are important for various processes such as neuronal integration, hormonal secretion, muscle contraction, and cardiac pace-making [6, 7]. In non-excitable cells, they are involved in cell proliferation, apoptosis, cell volume regulation, and lymphocyte differentiation [8–10].

Kv channels are homotetramers of α-subunits arranged around a central ion-conducting pore [11]. In addition to Kv α-subunits with an active ion-conducting ability, there are also electrically silent subfamilies, which are collectively referred to as electrically silent Kv (KvS) α-subunits [12, 13]. To date, there are four KvS subfamilies: Kv5, Kv6, Kv8, and Kv9. All KvS subunits do not form functional homotetramers, but form functional heterotetramers with the Kv2 subfamily, thereby, modulating the Kv2 current [12, 13]. Kv subunits have been implicated in cell-cycle progression, cell proliferation, and apoptosis [3, 14]. Regulation of these processes depends on both ion-conducting and non-conducting properties [14, 15]. In ion-conducting mechanisms, K^+^ channels can influence cell-cycle progression through cell volume regulation, modulation of membrane potential, and generation of driving force for intracellular Ca^2+^ [14]. In non-conducting mechanisms, it is through protein-protein interactions by the recruitment of signaling molecules that control cell proliferation [3, 14]. Many studies have shown that a specific Kv channel is important for cell proliferation [16–20]. However, mechanisms of Kv subunit-mediated tumor progression and invasion remain unclear.

While the physiological roles of Kv2/KvS heterotetramers in different tissues have been extensively studied [13], little is known about how KvS subunits are involved in cell proliferation and tumorigenesis. More importantly, it is unclear whether KvS subunits are involved in cancer development through either an ionic conduction mechanism, i.e., heterotetramerizing with Kv2.1, or a non-canonical mechanism, i.e., protein-protein interactions. In the present study, we analyzed the expression of KvS subfamilies and identified that KCNF1 (Kv5.1) was up-regulated in NSCLC. Down-regulation of KCNF1 in lung adenocarcinoma cell lines, A549 and H23 cells, reduced cell proliferation, migration, and tumor progression in mouse xenografts. NSCLC cells with silencing of KCNF1 exhibited non-transformed phenotypes with re-establishment of basement membrane integrity. We found that KCNF1 was primarily localized in the nucleoplasm in NSCLC cells and positively regulated ITGB4 expression. Our studies implicate that KCNF1 functions in the nucleus through a permeation-independent mechanism and promotes lung cancer by enhancing ITGB4 signaling.

## Results

### KCNF1 expression is increased in NSCLC

**Fig. 1:**
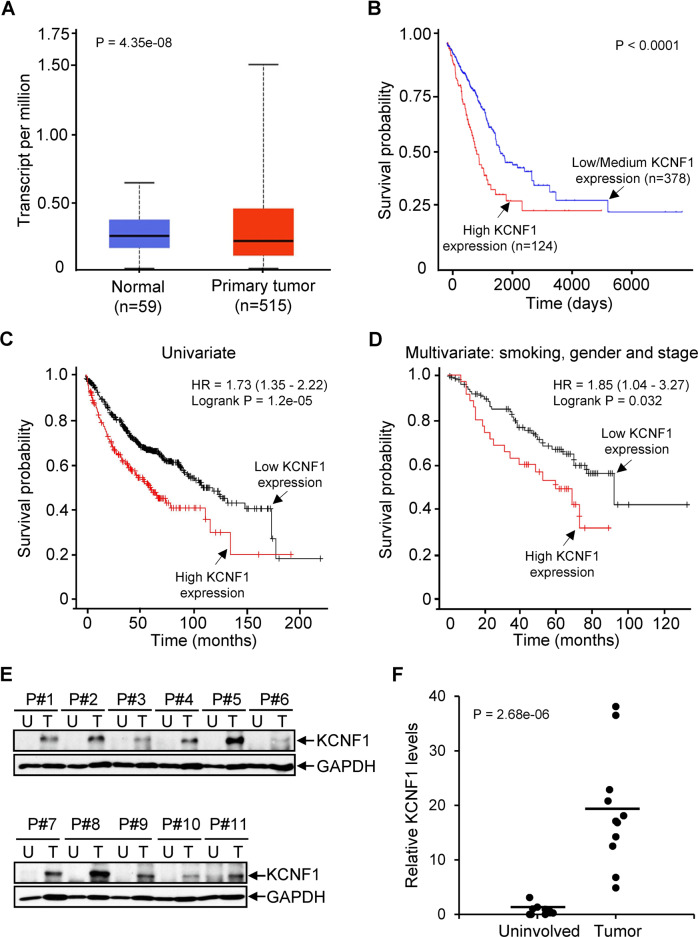
Expression of KCNF1 is elevated and associated with poor outcomes in NSCLC. **A** Analysis of TCGA dataset showed increased KCNF1expression in lung adenocarcinoma. **B** Kaplan–Meier survival analysis of TCGA lung adenocarcinoma dataset showed that increased expression of KCNF1 is correlated with poor survival. **C**, **D** Elevated KCNF1 expression is associated with reduced overall survival using univariate analysis and multivariate analysis after adjusting confounding factors including gender, AJCC stage N, and smoking using Kaplan–Meier Plotter. **E** Expression of KCNF1 in fresh frozen human lung cancer tissues and adjacent uninvolved lung tissues was analyzed by immunoblotting using anti-KCNF1 and anti-GAPDH. U uninvolved, T tumor. **F** Quantification of KCNF1 expression analyzed in (**E**).

To determine the role of KvS subfamilies in NSCLC, we explored the lung cancer datasets from The Cancer Genome Atlas (TCGA) to inquire expression levels of KCNF1 (Kv5.1), KCNG4 (Kv6.4), KCNV2 (Kv8.2), and KCNS1 (Kv9.1). Expression of KCNF1 and KCNV2 was significantly increased (Fig. 1A and Supplementary Fig. S1A), while expression of KCNS1 was decreased in lung adenocarcinoma (Supplementary Fig. S1B). The value for KCNG4 was too low to perform analysis. We next examined expression of the KvS subunits in relationship to survival of lung adenocarcinoma patients using UALCAN, an online portal for gene expression and survival analyses [21]. Patients with increased expression of KCNF1 had a significantly shortened survival; twofold higher survival in patients with low expression than patients with high expression (Fig. 1B). No significant differences in survival were observed for KCNV2 and KCNS1 (Supplementary Fig. S1C and S1D). We further explored whether KCNF1 is an independent prognostic factor in NSCLC using Kaplan–Meier Plotter [22]. The association of KCNF1 expression with overall patient survival was analyzed by both univariate and multivariate analyses from several independent datasets. We integrated all datasets and found that high KCNF1 expression was significantly associated with a poor survival by univariate analysis (HR = 1.73, 95% CI = 1.35–2.22, and P = 1.2e–5; Fig. 1C). After adjusting confounding factors including gender, AJCC stage N, and smoking, significant association of high KCNF1 expression with a poor survival was still detected (HR = 1.85, 95% CI = 1.04–3.27, and P = 0.032; Fig. 1D). To determine the clinical relevance of increased KCNF1 levels, we assessed its expression in fresh frozen lung tumor tissue samples. Tumor samples had a significant increase in KCNF1 expression compared to the matched uninvolved lung from the same patient determined by immunoblotting (Fig. 1E and F). These results indicate that KCNF1 is upregulated in NSCLC and is an independent prognostic biomarker analyzed by using several independent cohorts.

### Silencing of KCNF1 reduces cell proliferation, migration and tumor burden, and ectopic expression of KCNF1 increases cell proliferation

**Fig. 2:**
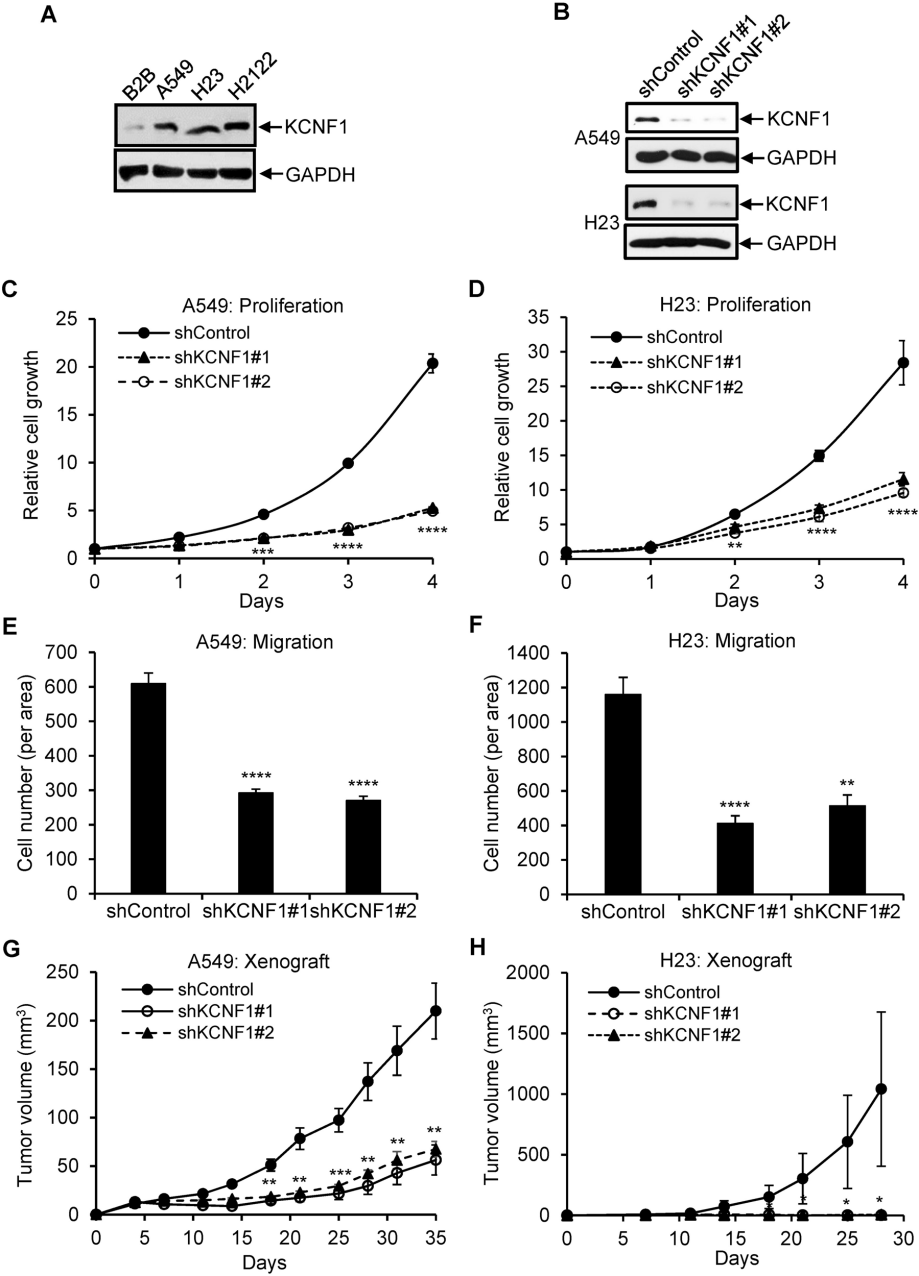
Silencing of KCNF1 reduces lung cancer tumorigenesis. **A** Lysates of Beas2B, A549, H23, and H2122 cells were analyzed by immunoblotting using anti-KCNF1 and anti-GAPDH antibodies. **B** Lysates of A549 and H23 cells transduced with lentiviral vectors expressing a control shRNA or two different shRNAs against KCNF1 were analyzed by anti-KCNF1 and anti-GAPDH. **C**, **D** Proliferation of transduced cells was analyzed. **E**, **F** Migration of transduced cells was analyzed. **G**, **H** Transduced cells were injected into the flanks of nude mice. Tumor volume was monitored. All data are mean ± SD. *P < 0.05; **P < 0.005; ***P < 0.0005; ****P < 0.00005.

**Fig. 3:**
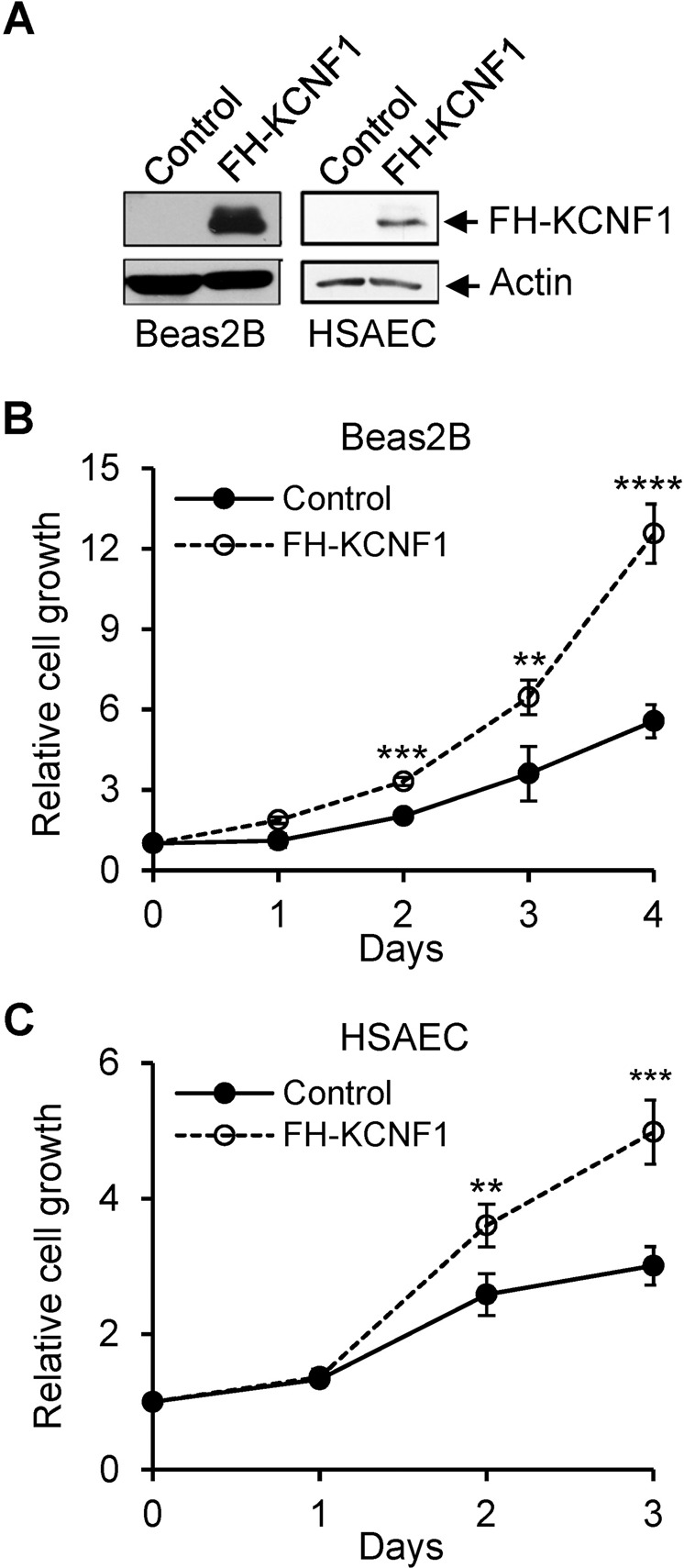
Ectopic expression of KCNF1 promotes cell proliferation. **A** Lysates of Beas2B and HSAEC cells transfected with a control vector or a vector expressing FLAG and HA double tags at the N-terminus (FH-KCNF1) were analyzed by anti-HA and anti-Actin. **B**, **C** Proliferation of transfected cells was analyzed. All data are mean ± SD. **P < 0.005; ***P < 0.0005; ****P < 0.00005.

To determine the biological function of KCNF1 in lung cancer, we first analyzed its expression in a non-transformed human lung epithelial cell line, Beas2B, and NSCLC cell lines, A549, H23, and H2122. All three NSCLC cell lines had higher KCNF1 expression than Beas2B cells (Fig. 2A). We silenced KCNF1 expression in both A549 and H23 cells by using lentiviral vectors expressing shRNAs that target KCNF1 (Fig. 2B). Silencing of KCNF1 using two different shRNAs significantly reduced proliferation and migration of both cell lines (Fig. 2C–F). Tumors in the flanks of mice injected using cells with silencing of KCNF1 grew significantly slower than those injected with control cells in nude mouse xenografts (Fig. 2G and H). We also examined whether KCNF1 knockdown affected the extent of apoptosis by analyzing the levels of cleaved caspases 3, 7, and 9, markers of programmed cell death. We found no differences in the levels of cleaved caspase 9 between control and KCNF1 knockdown in A549 and H23 cells and were unable to detect cleaved caspase 3 and caspase 7 (Supplementary Fig. 2). These results suggest that silencing of KCNF1 did not significantly affect apoptosis. We next expressed KCNF1 in non-transformed Beas2B and human small airway epithelial cells (HSAEC). Ectopic expression of KCNF1 in these two cell lines increased cell proliferation (Fig. 3). Altogether, these findings indicate that KCNF1 positively regulates cell growth, migration and tumorigenesis.

### Silencing of KCNF1 re-establishes basement membrane integrity

**Fig. 4:**
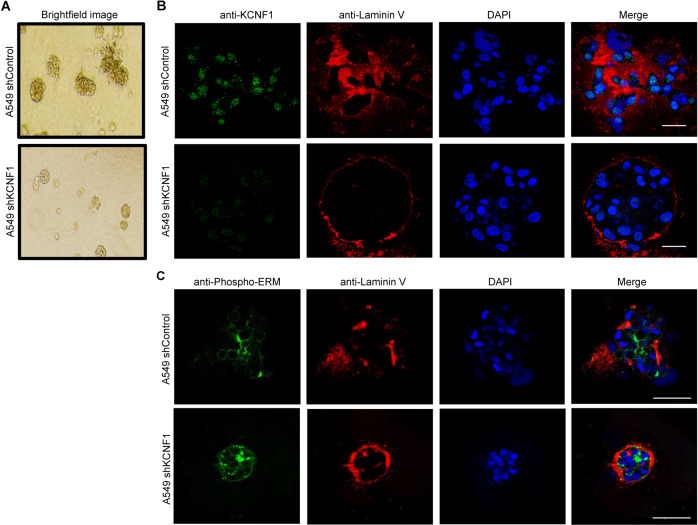
Silencing of KCNF1 re-establishes basement membrane integrity. **A** Bright field images of the spheroids from control A549 cells and A549 cells with knockdown of KCNF1. **B** Spheroids of control A549 cells and A549 cells with knockdown of KCNF1 were analyzed by IF using anti-KCNF1 and anti-laminin V antibodies. Cells were also stained with DAPI; scale bar, 20 μm at 40X objective. **C** Spheroids of control A549 cells and A549 cells with knockdown of KCNF1 were analyzed by IF using anti-phosphorylated ERM and anti-laminin V antibodies. Cells were also stained with DAPI; scale bar, 50 μm at 20X objective.

We examined whether KCNF1 influenced cellular structure and polarity and cell adherence, which are hallmarks of malignancy. Three-dimensional Matrigel cell culture allows for phenotypic differentiation of non-transformed cells from malignant cells. While non-transformed cells form highly organized spheroid-like structures, malignant cells on the contrary form unorganized and poorly formed structures. Strikingly, downregulation of KCNF1 in A549 cells induced a spheroid-like formation. By contrast, control A549 cells formed poorly differentiated structures, a characteristic of malignant cells (Fig. 4A). Immunofluorescence (IF) staining of the spheroids of A549 cells with KCNF1 knockdown showed enhanced polarized deposition of basement membrane compared to control cells as determined by laminin V staining, a basement membrane component (Fig. 4B). In addition, staining with antibodies against phosphorylated ezrin, radixin, and moesin (phospho-ERM) proteins, which induce formation and/or maintenance of spherical cell shape, also revealed increased basement membrane assembly with a more organized spheroid structure (Fig. 4C). These data indicate that abrogation of KCNF1 expression in a NSCLC cell line is sufficient to re-establish basement membrane integrity.

### KCNF1 is localized in the nucleoplasm and Golgi apparatus

**Fig. 5:**
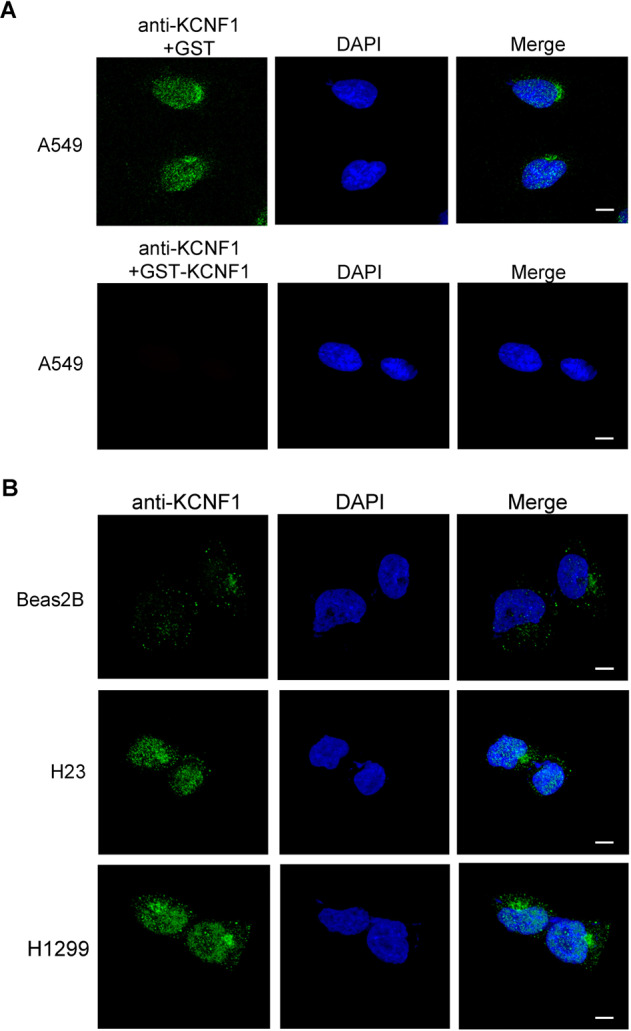
KCNF1 is primarily localized in the nucleoplasm of NSCLC cells. **A** A549 cells were stained with anti-KCNF1 in the presence of GST or GST-KCNF1 protein and DAPI, and analyzed by a confocal fluorescence microscope. **B** Beas2B, H23, and H1299 cells were stained with anti-KCNF1 and DAPI, and analyzed by a confocal fluorescence microscope. **A**, **B** scale bar, 5 μm at 63X objective.

It has been shown that KCNF1 forms a heterotetramer with Kv2.1 which is localized on the plasma membrane. To test this, we performed IF analysis on NSCLC cells, including A549, H23, and H1299, and non-transformed Beas2B cells to localize KCNF1. The anti-KCNF1 staining was specific as incubation of the antibody with recombinant GST-KCNF1, but not GST, completely removed the IF signals (Fig. 5A). Surprisingly, KCNF1 was primarily localized in the nucleoplasm and with some staining at the ER and/or Golgi apparatus in NSCLC cells (Fig. 5A and B). By contrast, while the signal was weak in Beas2B cells, KCNF1 was primarily present in the cytoplasm likely in the ER and/or Golgi apparatus (Fig. 5B). These data indicate that KCNF1 likely functions in the nucleus and appears to be independent of ion channel activity on the plasma membrane.

### Silencing of KCNF1 enhances cisplatin sensitivity

**Fig. 6:**
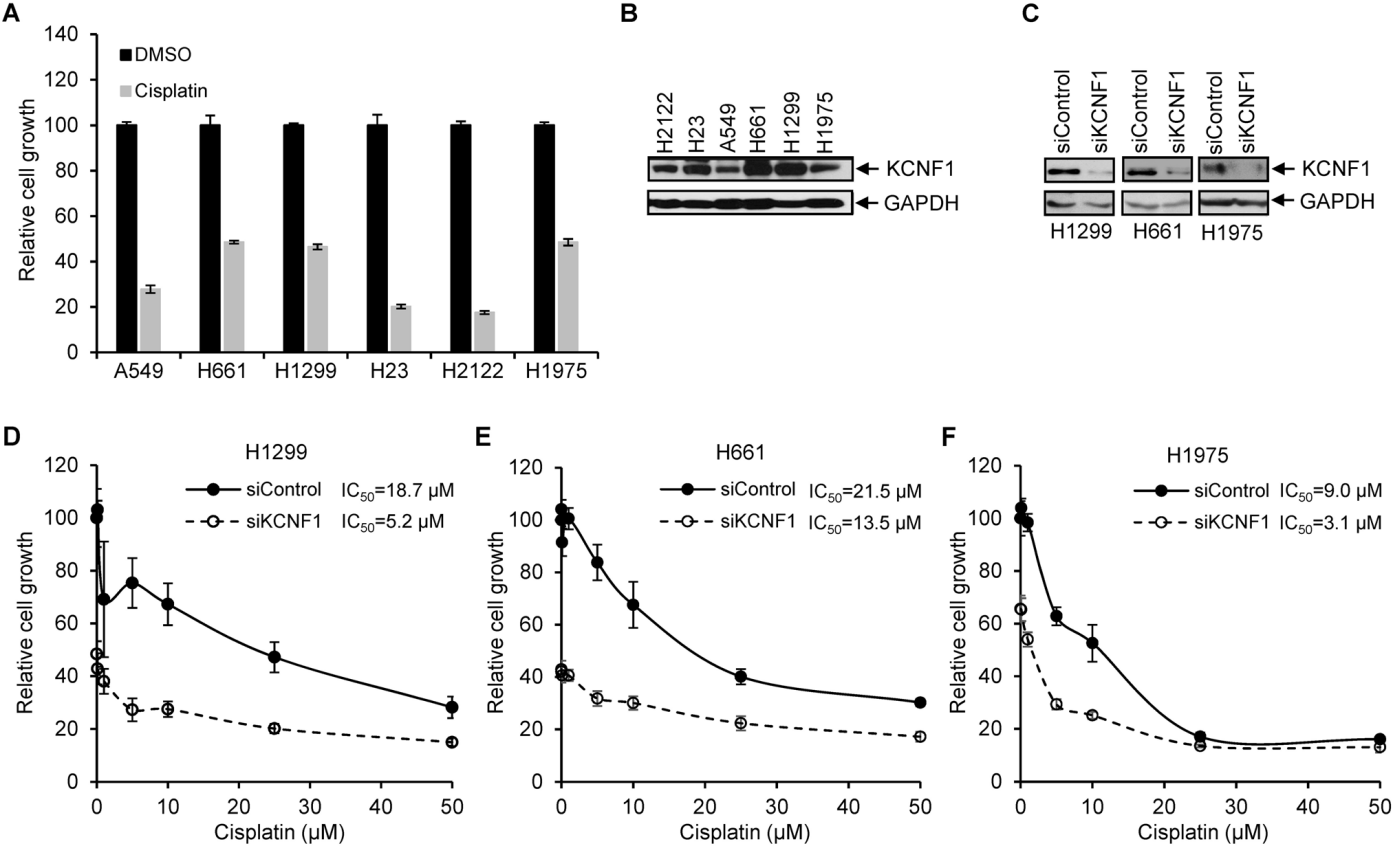
Silencing of KCNF1 increases cisplatin sensitivity. **A** NSCLC cell lines, A549, H661, H1299, H23, H2122, and H1975, were treated with DMSO or 10 μM cisplatin for 72 hours. Cell growth was analyzed by SRB staining. The cell growth with DMSO for each cell line was set at 100. **B** KCNF1 expression in NSCLC cell lines was analyzed by immunoblotting. **C** Lysates of H1299, H661, and H1975 transfected with a control siRNA or an siRNA targeting KCNF1 were analyzed by immunoblotting using anti-KCNF1 and anti-GAPDH. **D–F** H1299, H661, and H1975 cells were transfected with a control siRNA or an siRNA targeting KCNF1. Transfected cells were treated with different concentrations of cisplatin (0.01–50 μM) for 72 hours. Cell growth was analyzed by SRB staining. IC_50_ of each cell line transfected with siControl or siKCNF1 was calculated.

We examined whether silencing of KCNF1 could increase the sensitivity to cisplatin, the standard treatment for patients with advanced NSCLC. We first tested the sensitivity of various NSCLC cell lines to cisplatin and found that H661, H1299, and H1975 exhibited relatively higher resistance (Fig. 6A). Interestingly, higher KCNF1 expression was detected in H661 and H1299 cells (Fig. 6B). Knockdown of KCNF1 by siRNA increased cisplatin sensitivity in all three cell lines compared to control cells. The IC_50_ was decreased from 18.7 µM (treated with siControl) to 5.2 µM (treated with siKCNF1) in H1299, decreased from 21.5 µM to 13.5 µM in H661, and decreased from 9 µM to 3.1 µM in H1975 (Fig. 6D, E).

### KCNF1 is a regulator of ECM-integrin interactions and positively regulates ITGB4 downstream signaling

To define the mechanism by which KCNF1 elicits its tumor-promoting role, we performed microarray analysis using A549 cells transfected with either control or KCNF1 siRNAs. The scheme used for identifying KCNF1-dependent gene expression is shown in Supplementary Fig. S3A and described in the Materials and Methods section. First, a twofold change in gene expression with *P* value < 0.01 was applied to select genes with altered expression upon KCNF1 knockdown. Second, the selected genes were subjected to functional analysis to identify those involved in tumorigenesis and metastasis. Finally, these genes were further analyzed by qRT-PCR using a two-way comparison between A549 (control vs KCNF1 knockdown) and Beas2B (control vs KCNF1 overexpression). We identified the integrin β4 subunit (ITGB4) and laminin (LAMC1 and LAMC2) family, which are critical components of basement membrane, urokinase-type plasminogen activator (PLAU1), and hyaluronan mediated motility receptor (HMMR) as KCNF1-dependent genes (Supplementary Fig. S3A). Interestingly, all the identified genes are involved in ECM-receptor interactions and are known to play critical roles in tumor progression and metastasis [23–27]. We validated the expression of possible KCNF1 targets and examined expression of selected genes that are involved in ECM-integrin interactions (Supplementary Fig. S3B). We found that expression of ITGB4, LAMC1 and LAMC2 was reduced in cells with KCNF1 downregulation. Interestingly, expression of PLAU1 and HMMR1 was also reduced upon KCNF1 downregulation. By contrast, expression of integrin subunit α2 (ITGA2) was increased in cells with KCNF1 knockdown. No difference in expression of integrin subunit αV (ITGAV) was detected. These results suggest that KCNF1 is important for modifying cell-ECM interactions and plays a role in tumor progression and metastasis.

**Fig. 7:**
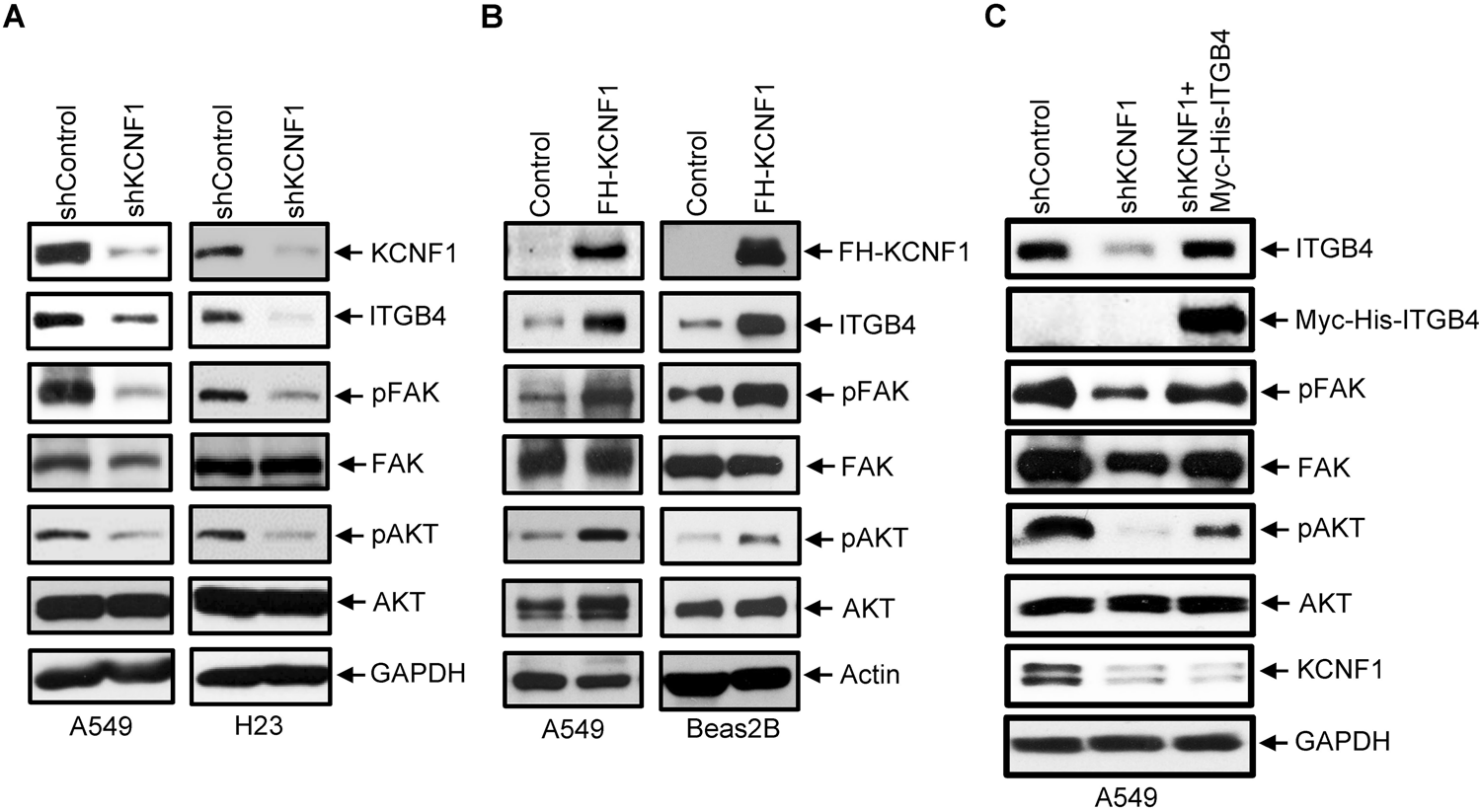
KCNF1 positively regulates ITGB4 expression and downstream signaling. **A** Lysates of A549 and H23 cells transduced with lentiviral vectors expressing a control shRNA or an shRNA against KCNF1 were analyzed by anti-KCNF1, anti-ITGB4, anti-p-FAK, anti-FAK, anti-p-AKT, anti-AKT, or anti-Actin. **B** Lysates of A549 and Beas2B cells with forced expression of FLAG and HA-tagged KCNF1 (FH-KCNF1) were analyzed by immunoblotting using anti-HA, anti-ITGB4, anti-p-FAK, anti-FAK, anti-p-Akt, anti-Akt, or anti-Actin. **C** A549 cells transduced with shControl or shKCNF1were transfected with a control vector or a vector expressing Myc-His-ITGB4. Levels of ITGB4, Myc-His-ITGB4, p-FAK, FAK, p-Akt, Akt, KCNF1, Actin, and GAPDH were analyzed by immunoblotting.

Given its prior association with malignant tumor formation [28], we further validated ITGB4 as a potential KCNF1 target. KCNF1 knockdown in NSCLC cells, A549 and H23, decreased ITGB4 expression and resulted in reduced phosphorylation of focal adhesion kinase FAK and AKT (Fig. 7A), which are downstream events enhanced by the cooperation between α6β4 integrin and oncogenic receptor tyrosine kinase [29, 30]. KCNF1 knockdown did not alter the expression of FAK and AKT in A549 and H23 cells compared to control cells (Fig. 7A). Conversely, expression of FLAG/HA-tagged KCNF1 in A549 cells and Beas2B cells augmented the levels of ITGB4 and phosphorylation of FAK and AKT, but not the levels of FAK and AKT, compared to control cells (Fig. 7B). To test whether the reduction of FAK and AKT phosphorylation was indeed resulting from reduced ITGB4 expression, we expressed Myc-tagged ITGB4 in A549 cells with KCNF1 knockdown. Ectopic expression of Myc-ITGB4 restored the levels of phosphorylation of FAK and AKT, but did not significantly alter the levels of FAK and AKT (Fig. 7C). These data suggest that KCNF1 positively regulates ITGB4 expression and promotes the downstream signaling events triggered by ITGB4 favoring tumor growth, invasiveness and metastasis.

## Discussion

Functions of Kv subunits in excitable cells have been studied in great detail, which include control of the resting potential and action potential duration [6]. Additionally, their roles in neoplastic cell proliferation have been recently assessed [3, 10]. Importantly, countless examples of aberrant expression of Kv channels in several types of cancer have been described [3, 10]. While Kv subfamilies can mediate cell-cycle regulation in an ion-conducting mechanism, experimental evidence has implicated that they are involved in cell proliferation through a permeation-independent, non-canonical mechanism [16–20]. Similarly, KvS subfamilies can modulate cell-cycle progression and cell proliferation through both mechanisms, i.e., forming heterotetramers with Kv2 subunits and interacting with cell cycle regulators through protein-protein interactions. A recent study implicated Kv9.3, a KvS subunit, in the proliferation of human colon and lung carcinoma cells [31]. While this study suggests a non-conducting role of Kv9.3, it is likely that the observed effects of reduced Kv9.3 expression on cell-cycle progression may also be caused by a change in available Kv2.1/Kv9.3 heterotetramers and Kv2.1 homotetramers.

Similar to other KvS subfamilies, KCNF1 is unable to form functional homotetramers, but is able to form heterotetramers with Kv2.1, whereby modulating its electrophysiological properties [32, 33]. The function of KCNF1 in cell proliferation and cancer progression has not been previously explored. In the present study, we demonstrated that silencing of KCNF1 in NSCLC cells reduced cell proliferation and tumor burden in mouse xenografts (Fig. 2). Although we cannot completely rule out that regulation of cell growth and cancer progression by KCNF1 relies on ionic conduction, our results implicate that it likely functions through a non-conducting mechanism as endogenous KCNF1 and exogenous KCNF1-EGFP were detected in the ER/Golgi and nucleoplasm, but not on the plasma membrane where Kv subunits normally function. However, how KCNF1 is localized and what it does in the nucleus are currently unclear. The N-terminal cytoplasmic tetramerization domain (T1) domain of Kv and KvS α-subunits required for tetramerization is also found in a subset of proteins containing the BTB/POZ domain [34]. The BTB domain was originally identified as a conserved motif present in the *Drosophila melanogaster* bric-à-brac, tramtrack and broad complex transcription regulators [35–37]. The BTB domains can function as a protein-protein interaction module that is able to both self-associate and interact with non-BTB proteins. Thus, the T1 domain of KCNF1 may interact with transcriptional regulators in the nucleus that modulate gene transcription. It is also interesting to understand how KCNF1, a multiple transmembrane domain containing protein which is normally synthesized and located in the ER lumen, ends up in the nucleoplasm. Identifying the cis-acting sequences within KCNF1 that regulate its localization and identifying factors associated with KCNF1 that help its translocation into the nucleus should further our understanding of the mechanisms leading to its nuclear localization and functions in gene regulation.

Microarray and gene function analysis identified several KCNF1 targets, including ITGB4 and laminin family (LAMC1 and LAMC2), involved ECM-integrin interactions. We further explored the effect of KCNF1 on ITGB4 and demonstrated that KCNF1 positively regulated ITGB4 expression and the downstream signaling (Fig. 7). These findings suggest that KCNF1 promotes the signaling mediated through the interactions between laminins and integrin β4 subunit. Integrins adhere to a variety of extracellular matrix components and signal multifarious functions such as survival, migration, polarity and differentiation [38, 39]. Interestingly, altered expression of integrins has been linked to cancer progression and metastasis [29, 40]. Previous findings demonstrated that ITGB4 promotes tumorigenesis and tumor progression. Integrin α6β4 is up-regulated in a number of tumors [28], which enhances multiple signal transduction cascades of RTKs, such as ErbB2, EGF-R and Met [30, 41, 42], thereby promoting cell proliferation, migration and invasion. Elevated expression of ITGB4 is associated with decreased overall survival in colorectal cancer [43] and non-small cell lung cancer [44, 45]. Silencing of ITGB4 in NSCLC cells reduced cell proliferation and increased cisplatin sensitivity [46]. Altogether, our results suggest that KCNF1 increases ITGB4 expression and signaling that disrupt cell adhesion and basement membrane integrity, thereby promoting cancer progression and metastasis.

Despite significant advancements in the treatment of lung cancer, the development of new approaches and therapies is necessary to increase the 5-year survival rate. Recent studies implicate K^+^ channels in neoplastic cell proliferation and the progression of cancer to a more malignant phenotype; highlighting them as excellent targets for cancer therapy [10, 47]. However, considering the importance of Kv subfamilies in activating nerve and cardiac action potentials, pharmacological targeting of ion channel activities might not be a viable approach, as they might present severe off-target effects. For example, whereas inhibition of Kv11.1 function or expression reduces the proliferation of tumor cells in vitro and in vivo [19, 48–51], drugs developed to inhibit Kv11.1 cause cardiotoxicity [52]. Thus, having the ability to specifically target electrically silent KCNF1 in lung cancer could provide the long-awaited significant advantage of not affecting normal and physiologically expressed K^+^ channels, but more precisely targeting cancer cells. An important question is how to target KCNF1 activity for NSCLC. As KCNF1 is overexpressed and located in the nucleus of NSCLC cells, understanding the mechanisms resulting in nuclear localization and identifying the nuclear factors, e.g., transcriptional regulators that are regulated by KCNF1, should provide a valid therapeutic approach to target KCNF1 activity in NSCLC.

## Materials and methods

### Animal studies

All animal experiments were approved by the Institutional Animal Care and Use Committee (IACUC). For subcutaneous xenografts, cells (2 × 10^6^ viable cells/flank) were injected subcutaneously into the lower flanks of 6-8-week female homozygous athymic nude mice (Jackson Laboratories, strain 002019) which were randomly assigned. Tumor growth was assessed weekly by caliper measurements and health of mice was closely monitored. Four to six weeks post implantation of cells, mice were sacrificed, and flank tumors were removed, imaged, and weighed. The data were not recorded blindly.

### Cell culture

Human non-transformed bronchial epithelial cell line, Beas2B, and NSCLC cell lines (A549, H23, H1299, H2122, H661, and H1975) were obtained from the American Type Culture Collection (ATCC). The cell lines were cultured in RPMI 1640 medium supplemented with 10% FBS and 1% penicillin-streptomycin in a humidified CO_2_ (5%) incubator at 37 °C. Non-transformed human small airway epithelial cells (HSAEC) were provided by Dr. John Minna (University of Texas Southwestern) and cultured in keratinocyte growth medium with supplements (KGM^TM^, LONZA). All cells were cultured, tested for mycoplasma, and passaged no more than 10 times for use in experiments.

### siRNA, shRNA, plasmid construct, and transfection

A control siRNA (#1027281) was purchased from Qiagen. siRNA targeting KCNF1 (sc-94734) was purchased from Santa Cruz Biotechnology. Lentiviral shRNAs targeting KCNF1 were purchased from Horizon Discovery. Human KCNF1 cDNA was amplified by PCR and subcloned into a pCDNA3 vector with a double FLAG and HA tags at the N-terminus. Human ITGB4 cDNA was subcloned into a pCMV vector with N-terminal Myc and His tags. Cells were transfected using Lipofectamine 2000 (Invitrogen) and analyzed 48 hours after transfection.

### Lentiviral vector production and infection

HEK293T cells (8 × 10^6^ cells) were seeded onto a 100-mm culture dish. Cells were transfected 16 hours later with a lentiviral vector expressing shRNA (12 µg), pMD.G (2 µg), and psPAX2 (8 µg) using Lipofectamine 2000. Medium was replaced with fresh growth medium (7 ml) containing sodium butyrate (10 mM) 16 hours later. Medium containing lentivirus was collected 24 hours later, centrifuged, filtered using a 0.45 µm syringe filter, and saved at −80^o^C. Cells were further incubated in fresh growth medium (7 ml) containing sodium butyrate (5 mM), and medium was collected 24 hours later, filtered, and saved at −80 ^o^C. To transduce cells with lentiviral vectors, cells (3 × 10^5^ cells/well) were seeded onto a 6-well dish and incubated with 1 ml of growth medium containing lentivirus and polybrene (8 µg/ml) for 16 hours. After which, medium was replaced with fresh growth medium. 48 hours after transduction, cells were selected with puromycin (1–2 µg/ml) for 7 days.

### Cell proliferation and migration assays

Cell proliferation was measured by Sulforhodamine B (SRB) assays as previously described [53]. Briefly, cells (1,000–2,000 cells/well) were cultured in a 96-well plate and harvested at different time points. One 96-well plate was harvested 6 hours after plating, which was considered as time 0. Cells were fixed with 10% trichloroacetic acid for 30 min at 4 ^o^C and subjected to SRB staining. Cell growth rate was calculated by normalizing the readings of each time point with 0 time point, which was set at 1, to control for plating of equal number of cells among different treatments. To analyze the effect of KCNF1 silencing on cisplatin sensitivity, cell growth was measured 3 days after the addition of 8 different concentrations of cisplatin. To calculate IC_50_, the percentage of cell growth for each concentration relative to no cisplatin was obtained and plotted using PRISM. IC_50_ was calculated by an equation: Y = (% of cell growth with the highest concentration) + (% of cell growth without cisplatin - % of cell growth with the highest concentration)/(1 + (X/IC_50_)), in which X = cisplatin concentration and Y = % of cell growth. The final IC_50_ value and standard error was calculated by using non-linear regression to fit the raw data using PRISM software. To assess cell migration, 15,000-30,000 cells in serum-free media were seeded into trans-well inserts (Corning) containing 8-μm permeable pores and allowed to migrate toward 10% FBS complete media. Inserts were washed with PBS three times to remove debris and migrated cells on the bottom of the inserts were fixed with 2% glutaraldehyde solution followed by crystal violet (2%) staining for 1 hour at room temperature. Images were taken using an inverted microscope where ten independent fields were counted for each trans-well and the average number of cells per field was presented in a graph.

### 3-D cell culture and immunofluorescence analysis

Cells were grown in growth factor-reduced Matrigel (BD Biosciences, 356231) as described [54]. 2,000 cells were seeded per well chamber and grown in a 4% Matrigel basement membrane supplemented with EGF on top of a 100% Matrigel layer. At 5–8 days, images of the colonies were captured using an inverted microscope and analyzed by determining the number of spheroids and aggregates. For immunofluorescence analysis of 3-D cell culture, cultures were fixed with 2% paraformaldehyde for 20 min and permeabilized with 1X PBS containing 0.5% Triton X-100. Primary antibodies against Laminin V (Santa Cruz Biotechnology, sc13587), KCNF1 (Sigma, HPA014738), and phosphor-ERM (ezrin/radixin/moesin; Cell Signaling, 3141) were used at 1:250 dilution. After overnight incubation at 4 °C, Alexa Fluor 488- (Jackson ImmunoResearch, 111-545-003) or Cy3- (Jackson ImmunoResearch, 115-165-003) conjugated secondary antibodies were added at 1:500 dilution and incubated for 50 min at room temperature. Cells were rinsed several times with 1X PBS and mounted with Vectashield mounting medium with DAPI (Vector Laboratories, Inc., H1200).

### Microarray analysis and identification of KCNF1 downstream targets

RNA samples (*n* = 3) isolated from A549 transfected with siControl or siKCNF1 were subjected to microarray analysis. Differential gene expression was analyzed using a cut-off of twofold difference and *P* < 0.01. Using this cut-off, 546 genes were obtained with altered expression upon KCNF1 knockdown. These genes were further analyzed using WEB-Based Gene Set Analysis Toolkit to identify those involved in neoplastic processes including carcinogenesis, adhesion, and invasiveness, ECM-receptor interactions, and NSCLC. Overall, 20 genes were identified to be involved in these processes. These genes were further validated with qRT-PCR using RNA samples from A549 cells transfected with siControl or siKCNF1 as well as from Beas2B cells transfected with a control vector or a vector expressing HA-KCNF1. The genes both downregulated in A549 cells transfected with siKCNF1 and upregulated in Beas2B cells transfected with HA-KCNF1 were identified and shown in supplementary Fig. S3A.

### RNA isolation and quantitative reverse transcription-polymerase chain reaction (qRT-PCR) analysis

Total RNA was extracted using TRIzol Reagent (Invitrogen). 3 μg of RNA was reverse-transcribed using random primers. Real-time PCR was performed using ROX Free Real Time PCR Mastermix (Denville Scientific, Inc.) and the Bio-Rad CFX96 qPCR detection system.

### Immunoblot analysis

Protein extracts from NSCLC cell lines and fresh frozen lung tissue samples were prepared in lysis buffer (0.5% Triton X-100, 50 mM β-glycerophosphate, pH 7.2, 0.1 mM dithiothreitol, 2 µg/mL leupeptin, and 2 µg/mL aprotinin). Extracts were resolved on 10% SDS-PAGE gels and transferred onto nitrocellulose membranes. Membranes were blocked in Tris-buffered saline (TTBS) containing 10 mM Tris-Cl (pH 7.4), 140 mM NaCl, 0.1% Tween 20, and 3% nonfat dry milk for 1 hour and incubated with TTBS containing indicated antibodies at 0.5 µg/mL for 12–16 hours at 4 ^o^C. The following antibodies were used for immunoblotting: anti-KCNF1 (Sigma, HPA014738), anti-ITGB4 (Abcam, ab182120), anti-HA (Cell Signaling, 2367), anti-His (Proteintech, 10001-0-AP), anti-phospho-FAK (Tyr397, Invitrogen, 44624 G), anti-FAK (Invitrogen, AHO0502), anti-phospho-AKT (pS473, Cell Signaling, 9271), anti-AKT (Cell Signaling, 9272), anti-caspase 3, -caspase 7, and -caspase 9 (Cell Signaling: 9662, 9492, 9508), anti-GAPDH (Cell Signaling, 5174), and anti-Actin (Sigma, A2066). The membranes were extensively washed in TTBS and bound antibodies were visualized with horseradish peroxidase (HRP)-coupled secondary antibodies and ECL western blotting detection reagent (Amersham, RPN2106).

### Human tissue samples

Frozen lung tumor tissue samples were obtained from The Lung Cancer Biospecimen Resource Network (LCBRN, University of Virginia).

### Data analysis

Data were collected from at least three independent, replicate experiments that were performed on separate cultures and separate occasions. All data are presented as the mean ± SD.

Since our studies are based on normal distribution, statistical significance (*P* value) was calculated by unpaired two-tailed Student’s *t* -test or one-way ANOVA. For xenograft studies, sample size was estimated by using G*Power 3.1.5 software (Heinrich-Heine University, Germany) using following parameters: One-Way ANOVA F-test (f = 0.5), α error probability = 0.05, power (1- β-error probability) = 0.80, and effect size = 0.8 since a large effect is expected.

## Supplementary information

Supplemental Material

## Data Availability

All data generated or analyzed during this study are included in this published article and its supplementary information files. The datasets are also available from the corresponding author on reasonable request.
