# Supplementary material for: KCNF1 promotes lung cancer by modulating ITGB4 expression
Source: Cancer Gene Ther. 2022 Nov 16;30(3):414–23. doi: 10.1038/s41417-022-00560-4 (PMC10014577; doi:10.1038/s41417-022-00560-4)
Supplement: Supplementary file 1 — Supplemental Material [file 41417_2022_560_MOESM1_ESM.pdf]

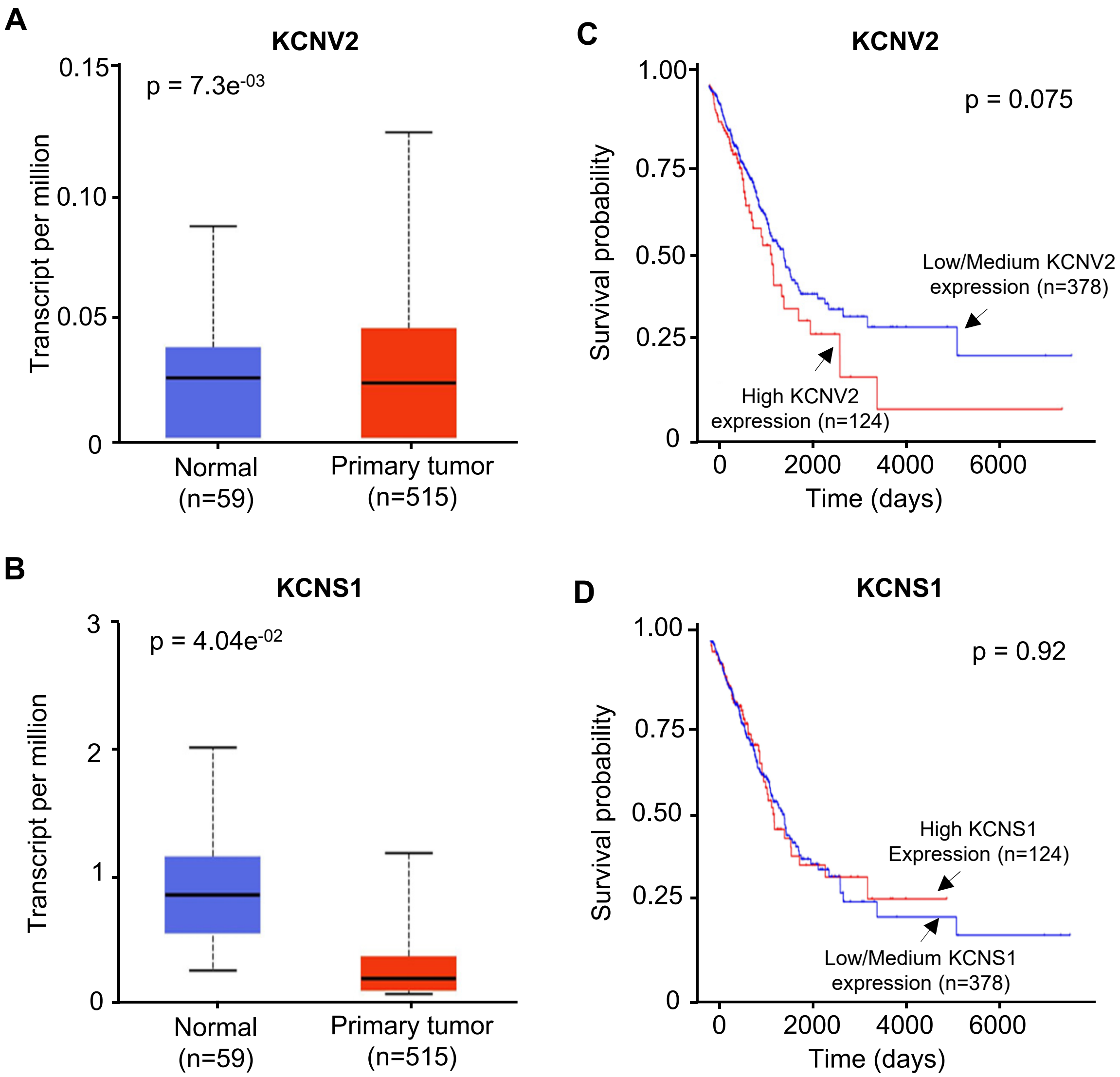

Supplementary Fig. 1 Expression of KCNV2 and KCNS1 in lung adenocarcinoma and their association with survival. A-B Expression of KCNV2 and KCNS1 in lung adenocarcinoma analyzed using TCGA dataset. **C-D** Association of KCNV2 and KCNS1 expression with survival by Kaplan-Meier survival analysis using TCGA dataset.

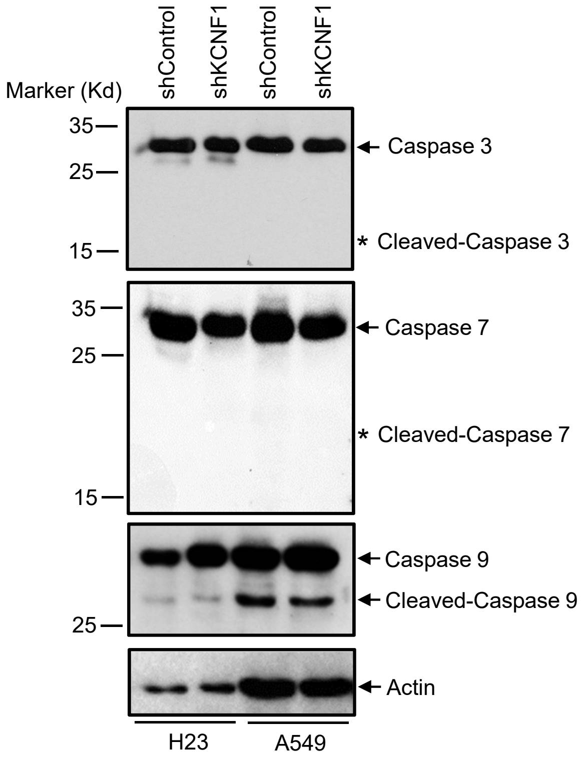

Supplementary Fig. 2 No significant changes in the levels of cleaved caspase 3, caspase 7, and caspase 9 in H23 and A549 cells with KCNF1 knockdown. Extracts of control H23 cells, H23 cells with KCNF1 knockdown, control A549 cells, and A549 cells with KCNF1 knockdown were analyzed by immunoblotting with anti-caspase 3, anti-caspase 7, anti-caspase 9, or anti-Actin antibodies. Full-length caspases and cleaved caspases are indicated. The positions of cleaved caspase 3 and caspase 7, which were not detected, are indicated by asterisks.

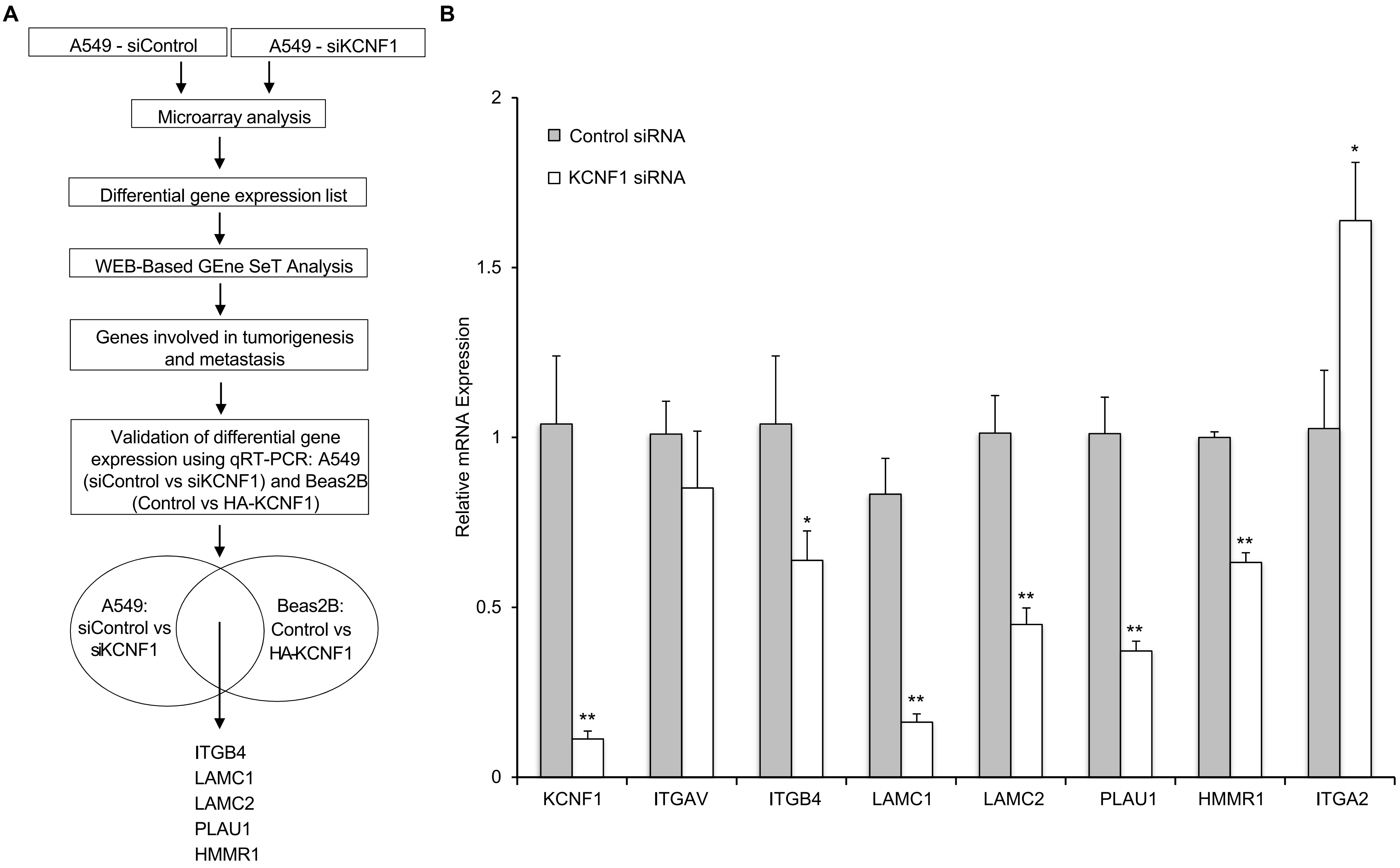

Supplementary Fig. 3 Identification of KCNF1 targets that regulate ECM-receptor interactions. A Schematic illustration of the approaches for identifying KCNF1 targets. **B** Expression of KCNF1, ITGAV, ITGB4, LAMC1, LAMC2, PLAU1, HMMR1, and ITGA2 in A549 cells transfected with a control siRNA or KCNF1 siRNA was analyzed by qRT-PCR. Data are mean ± SD. *, $P < 0.05$, **, $P < 0.01$
